# A novel, non-invasive approach to esophageal coin removal in a child using oral olive oil: case report

**DOI:** 10.3389/fped.2026.1936681

**Published:** 2026-09-04

**Authors:** A. Y. Akkam, L. A. Albakkour, N. S. Alkhudhairy, A. Imtiaz

**Affiliations:** 1 Pediatric Emergency Department, Children's Hospital, King Saud Medical City, Riyadh, Saudi Arabia; 2 Pediatric Department, Prince Sultan Military Medical City, Riyadh, Saudi Arabia

**Keywords:** coin, esophageal foreign body, non-invasive, olive oil, pediatric emergency

## Abstract

**Introduction:**

Foreign body ingestion is a common reason for pediatric emergency department presentation, with coins representing a frequently encountered ingested object. Clinical presentation ranges from being entirely asymptomatic to symptoms such as drooling, dysphagia, cough and chest discomfort. Failure to promptly recognize and appropriately manage an esophageal foreign body may result in serious complications, underscoring the need for a timely diagnosis and management.

**Case presentation:**

We report the case of a 6-year-old boy who presented with drooling and retrosternal pain, approximately 4 h after accidentally swallowing a coin. Plain radiographs demonstrated a round radio-opaque foreign body lodged within the distal esophagus. Following an informed discussion with the parents, instead of immediate endoscopic retrieval, a conservative trial consisting of oral olive oil (1 mL/kg) followed by unrestricted water intake was undertaken. Repeat radiographic evaluation one hour later demonstrated complete passage of the coin beyond the esophagus to the ileocecal region, with complete resolution of the child's symptoms. He was discharged home from the emergency department in a stable condition. The coin was passed spontaneously in the stool within 24 h, without any complications.

**Conclusion:**

This case serves as a hypothesis generating concept of the potential role of oral olive oil as a simple, inexpensive, minimally invasive adjunct for selected cohort of children with coins lodged in the lower esophagus. Such an approach may reduce the need for endoscopic intervention, minimize exposure to anesthesia, shorten emergency department length of stay, reduce rates of admission for endoscopy, and decrease healthcare resource utilization. Nevertheless, patient selection should be done cautiously, with strict adherence to established foreign body ingestion management.

## Introduction

Foreign body ingestion is amongst the common reasons for pediatric emergency department (ED) attendance, and complications due to objects retained in the upper gastrointestinal tract (GIT) or aerodigestive tract frequently necessitate prompt radiographic evaluation and specialist intervention (1, 2). Coins have been reported to be the most common ingested foreign body amongst children, exceeding accidental ingestions of batteries, fish bones, metallic objects, magnets, etc (3). The highest incidence is amongst children between six months to six years of age, with a modest male predominance reported (1, 3). Clinical presentations vary widely, with some children remaining entirely asymptomatic, while others present with vomiting, dysphagia, drooling, cough, discomfort in the throat or chest, with a few children developing respiratory symptoms (1, 3). Data from the American Association of Poison Control Centers recorded 66,519 cases of foreign body ingestion in children under five years of age in a single reporting year (4). In the Kingdom of Saudi Arabia specifically, the annual incidence of pediatric rigid esophagoscopy for foreign body removal rose more than five folds, after the introduction of a new set of coin currencies between 2016 and 2017, an increase almost entirely attributable to increase in coin ingestion (5).

Foreign bodies cause significant morbidity in less than 1 percent of all cases. In the United States, esophageal foreign body occlusion has been estimated to contribute to approximately 1,500 deaths per year (2). Most ingested foreign bodies pass through the GIT without an incident, with only 10–20 percent requiring any intervention, and less than one percent requiring any surgical intervention (2). Since roughly 40 percent of pediatric ingestions are unwitnessed, diagnosis can be delayed (6). Therefore, a high index of clinical suspicion, a reliable history and appropriate radiographic evaluation are central to a timely diagnosis and favorable outcome (7).

Current management guidelines differ on how asymptomatic or minimally symptomatic esophageal coins should be handled (8, 9). Management approaches that avoid unnecessary sedation, anesthesia and endoscopy are of particular interest in pediatric practice. In this case report, we describe the use of oral olive oil to facilitate the spontaneous passage of a coin lodged in lower esophagus, an approach that has not been previously reported in pediatric literature to the best of our knowledge, based on the review of databases such as PUBMED, OVID, Google Scholar.

## Case description

A previously healthy 6-year-old boy with no prior surgical history was brought by his parents to the pediatric emergency department at King Saud Medical City, Riyadh, Kingdom of Saudi Arabia, four hours after accidentally swallowing a foreign object. The incident was witnessed by parents. He presented with sudden onset of drooling and central chest pain. He had no stridor, hematemesis, dysphagia, odynophagia, vomiting or cough.

On examination, his vitals were within normal limits for his age. He appeared calm, showed no signs of respiratory distress and maintained normal oxygen saturation on room air. Drooling was noted on general inspection. Neurological, cardiovascular, respiratory and abdominal examinations were otherwise unremarkable.

## Diagnostic assessment

**Figure 1:**
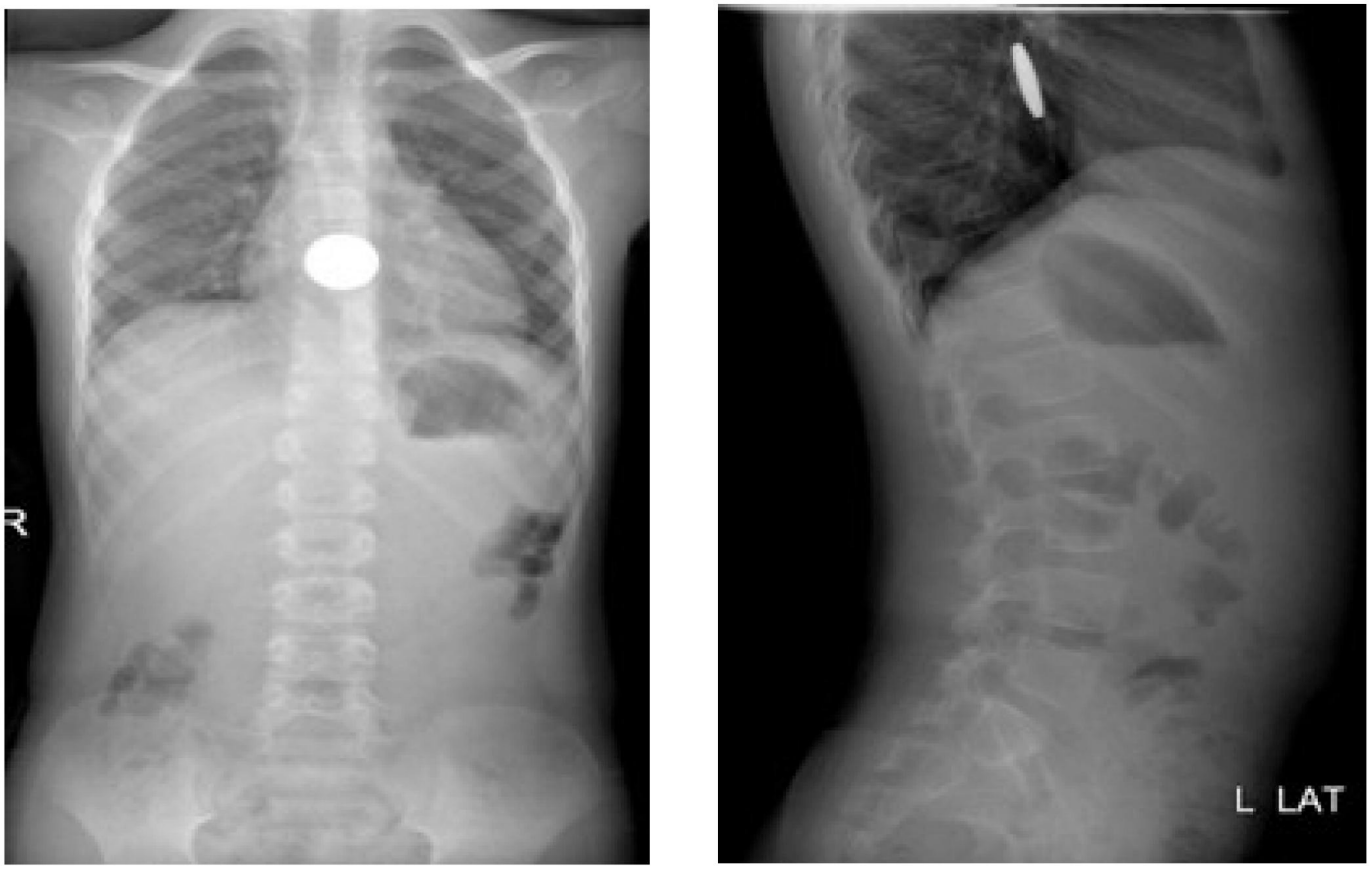
Initial posterior-anterior **(A)** and lateral **(B)** radiographs showing a radio-opaque round object lodged in the lower third of esophagus.

Posterior-Anterior and lateral radiographs of the chest and abdomen were obtained, which demonstrated a circular radio-opaque shadow in the lower third of the esophagus. The coronal orientation on the frontal projection and posterior position on the lateral view were consistent with esophageal impaction. The absence of a double rim sign further supported the foreign body to be a coin, rather than a button battery, confirming the diagnosis of an ingested coin lodged in the distal esophagus (Figure 1).

The clinical presentation of acute drooling and chest pain following a witnessed ingestion event, together with a radio-opaque shadow on chest and abdominal radiography, was most consistent with a foreign body, likely a coin, impacted in the distal esophagus. Other considerations in a child who presents with drooling, chest discomfort and possible foreign body ingestion include foreign body of a different composition such as a button battery or magnet, food bolus impaction, or a trachea-bronchial foreign body, eosinophilic esophagitis. Although with the sudden onset of symptoms, history of witnessed coin ingestion, a medically and surgically free course, and the radiographic appearance of the foreign body make the other differentials less likely.

## Treatment and outcome

Following confirmation of the diagnosis, management options were discussed extensively with the patient's parents, including the anticipated benefits and potential limitations of a conservative trial, the alternative option of endoscopic retrieval under general anesthesia. After obtaining parental consent and confirming the absence of any contra-indications, a non-invasive therapeutic trial was undertaken. The patient was given oral olive oil at a dose of 1 mL/kg, followed immediately by unrestricted oral water intake (as tolerated by the child). The patient was supported by putting him in a semi-sitting position, continuous cardiorespiratory monitoring, pulse oximetry and observation for any vomiting. Since the intervention took place in a pediatric ED setting, suction equipment and pediatric airway management were immediately available in case of any deterioration. Throughout the 1-hour observation period, the patient remained vitally stable with no aspiration, worsening of the pain or respiratory compromise.

**Figure 2:**
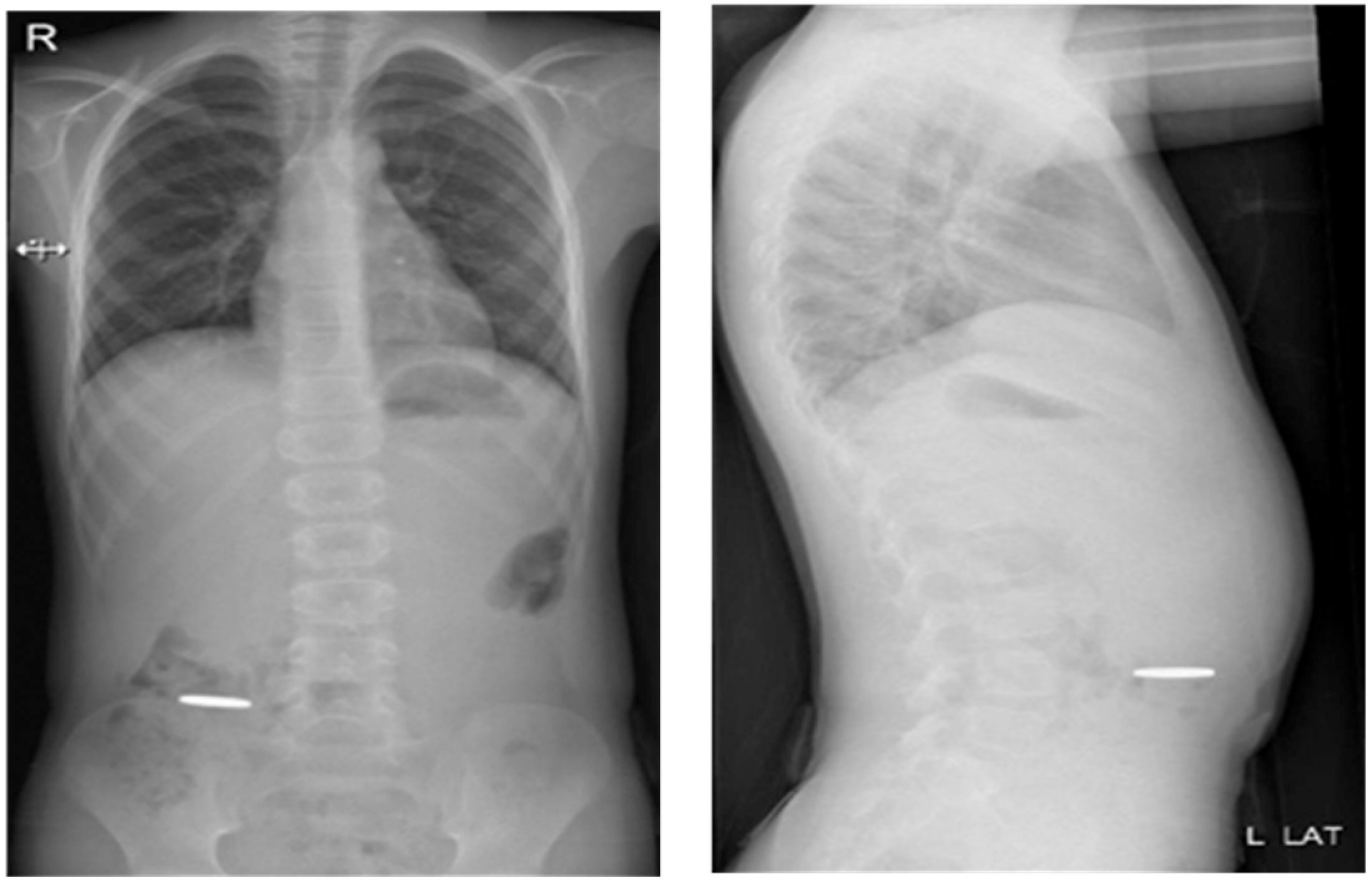
Follow-up posterior-anterior **(A)** and lateral **(B)** radiographs obtained one hour after the intervention showing the foreign body at the ilieo-cecal junction.

Approximately 1 h after the administration of olive oil, the child reported complete resolution of chest discomfort, drooling had resolved. Radiographs were repeated immediately after the resolution of symptoms, approximately 1 h after olive oil administration, to determine the location of the foreign body (Figure 2).

The patient was discharged home in a stable condition, approximately one hour after the successful olive oil trial. Before discharge, he was tolerating oral intake well, with no odynophagia, dysphagia or vomiting with normal vital signs. His parents were instructed to observe his stool for passage of the coin and to return within four days, or sooner if any complications arose. The coin was passed uneventfully in the stool within approximately 24 h of discharge. The family was also counseled on safe storage of small household items and on close supervision of young children during play, to reduce the risk of recurrence.

Informed consent was obtained from the patient's parents for the case to be reported and published for educational purposes. All identifying details have been removed while reporting the case. Formal ethics committee approval was waived, consistent with local policy.

## Discussion

Esophageal foreign body impaction is a common pediatric emergency, presenting with a wide range of symptoms. The specific symptoms depend on the type and location of the object and age of the child (2). Amongst the various foreign bodies, coins have been identified to be the most common (3). An impacted esophageal coin typically produces acute dysphagia, chest discomfort, drooling, reduced appetite, vomiting, hematemesis, or a foreign body sensation, although a proportion of children remain asymptomatic (10, 11). Complications, if they occur, include abscess formation, bleeding, perforation, intestinal obstruction, and respiratory distress from tracheal compression due to peri-esophageal soft tissue edema (11, 12).

Management guidelines for esophageal foreign body ingestion in children differs internationally. The European Society for Pediatric Gastroenterology, Hepatology and Nutrition (ESPGHAN) recommends endoscopic removal in every case, whereas the North American Society for Pediatric Gastroenterology, Hepatology and Nutrition (NASPGHAN) recommends urgent endoscopic removal for symptomatic patients and a period of observation of 12–24 h for asymptomatic patients, with endoscopic removal for those patients in whom the foreign body fails to pass spontaneously within the observation window (8, 9). Since the guidelines differ, clinical judgement plays a central role when managing stable children with distal esophageal foreign bodies. Most coins that reach the stomach spontaneously do not require emergency endoscopic removal, and pass the gastrointestinal tract within one to two weeks (1, 10).

In a retrospective cohort of children with esophageal impaction of coin, spontaneous passage to stomach occurred in 28% of the cases that can be classified as low risk, with a mean time to passage of 5 h, and a range of 2.3–12 h (13). This was in contrast to high-risk patients in whom the spontaneous passage did not occur at all. The child in this report was carefully selected, so as to be a low-risk patient, and passed the coin within one hour of olive oil administration, though spontaneous passage cannot be ruled out. The Royal Children's Hospital of Melbourne clinical practice guidelines describe high-risk foreign bodies to be button batteries lodged in esophagus, magnet plus a metal object or more than one magnet, sharp objects, lead based objects, large objects, expandable foreign bodies, objects impacted in oropharynx (20). High-risk patients have been described as those with pre-existing GIT abnormalities, neuromuscular disease and eosinophilic esophagitis (20).

A review of the existing literature on management of ingested pediatric foreign bodies demonstrates that most published studies are retrospective cohort studies or institutional case series (1, 11, 12). Likewise, the clinical recommendations are largely derived from expert consensus informed by observational evidence rather than randomized controlled trials. As a result, variation amongst clinicians in clinical practice persists, particularly for minimally symptomatic or asymptomatic children with distal esophageal coin impaction. Even amongst children who ultimately require endoscopic removal, the precise timing is debated (8, 9). A retrospective comparison of daytime vs. overnight esophageal foreign body removal, by Esparaz et al, suggested that a period of clinical observation may not compromise safety. In certain cases where endoscopic retrieval is necessary, standard grasping instruments can fail with large, smooth, or spherical objects, and adapted techniques or instruments have to be used (14). Access to timely diagnosis and endoscopic care for pediatric esophageal foreign bodies is not uniform, varying based on socioeconomic and geographical differences. This underscores that the management pathways should include simple and low cost, non-invasive adjuncts such as the one described in this report. Experimental, *in-vitro* and *in vivo* studies have demonstrated the benefit of honey and honey combined with olive oil (mixed in a 1: 1 ratio) in cases of esophageal button batteries (15, 16). These findings have subsequently been incorporated into the international management guidelines as a temporary measure while urgent endoscopy is arranged. The recommended dose of honey to be used in such cases is 10 mL, given in a maximum of 6 repeated doses (9). This is in consideration with the fact that the risk of aspiration with emergency vs. elective anesthesia cases is similar (17). The dose of olive oil used in our case was an empirical dose, with the intention of using a small volume to avoid the risk of aspiration, if the patient needed definitive management with endoscopic removal.

Use of endoscopic instillation of Olive Oil for therapeutic upper GI endoscopy in an adult patient has been described by Schneider at. Al. for treatment of an impacted food bolus (18). This is in contrast with our report where olive oil was administered as a non-invasive intervention, orally, in a pediatric patient with distal esophageal coin impaction, to avoid endoscopic retrieval altogether. Furthermore, food impaction often occurs in the setting of underlying esophageal pathology, and differs mechanistically from the impaction of an inert blunt metallic foreign body in an otherwise healthy child (19).

This case adds to the proof-of-concept that, in selected children with a coin lodged in the distal esophagus, a trial of oral olive oil may serve as a safe intervention to help in spontaneous passage, thereby avoiding endoscopic retrieval and exposure to general anesthesia. Although spontaneous migration cannot be excluded as an alternative explanation, the close temporal relationship between olive oil administration and progression of radiographic findings raises the possibility that lubrication may have contributed to passage. To our knowledge, no published pediatric case report has previously described oral olive oil as a conservative adjunct for distal esophageal coin impaction, making this observation novel and hypothesis generating. An important strength of this approach lies in its potential to reduce exposure to invasive procedures, reduce administration of general anesthesia and rates of hospital admission. Successful management in the ED may also reduce ED length of stay, procedural costs and hospital resource utilization. The clinician must, however, maintain a low threshold for proceeding to endoscopic removal whenever clinical deterioration occurs or spontaneous passage fails. There were several limitations of the management and reporting of this case. This report describes a single patient, and spontaneous passage cannot be ruled out. The optimal dose or optimal timing for the administration of oral olive oil is also not known. The lubrication provided by olive oil as the proposed mechanism of action is hypothetical, and has not been investigated experimentally. This management approach is limited to coin ingestion, and cannot be extrapolated to high-risk foreign bodies or high-risk patients. Prospective multicenter studies, such as randomized controlled trials, are needed to evaluate the safety, feasibility, optimal dosing strategy and comparative effectiveness of oral olive oil in distal esophageal foreign body retrieval amongst pediatric patients.

## Conclusion

This case report describes the successful spontaneous passage of radiographically confirmed distal esophageal coin, shortly after the administration of oral olive oil in a clinically stable child. Although a causal relationship cannot be established from a single observation, neither can the spontaneous passage of the coin be ruled out, the temporal association between intervention and rapid migration of coin suggests that oral olive oil may serve as a non-invasive adjunct when dealing with low-risk patients and low-risk foreign bodies.

To our knowledge, this is the first reported pediatric case describing use of oral olive oil for non-invasive retrieval of distal esophageal coin impaction. Therefore, the findings of this report should be interpreted as proof of concept rather than evidence of therapeutic efficacy. This report also underscores the importance of individualized, patient centered decision making in pediatric foreign body management. We recommend a randomized controlled trial to establish causality and determine efficacy of this intervention in low risk patients with distal esophageal foreign bodies.

## Data Availability

The original contributions presented in the study are included in the article/Supplementary Material, further inquiries can be directed to the corresponding author.
